# Personalized Prediction of Kidney Function Decline and Network Analysis of the Risk Factors after Kidney Transplantation Using Nationwide Cohort Data

**DOI:** 10.3390/jcm11051259

**Published:** 2022-02-25

**Authors:** Moongi Simon Hong, Yu-Ho Lee, Jin-Min Kong, Oh-Jung Kwon, Cheol-Woong Jung, Jaeseok Yang, Myoung-Soo Kim, Hyun-Wook Han, Sang-Min Nam, Korean Organ Transplantation Registry Study Group

**Affiliations:** 1 Department of Biomedical Informatics, CHA University School of Medicine, CHA University, Seongnam 13488, Korea; moongi@chauniv.ac.kr; 2 Institute for Biomedical Informatics, CHA University School of Medicine, CHA University, Seongnam 13488, Korea; 3 Division of Nephrology, Department of Internal Medicine, CHA Bundang Medical Center, CHA University, Seongnam 13496, Korea; borywork@chamc.co.kr; 4 Department of Nephrology, BHS Hanseo Hospital, Busan 48253, Korea; drkongj@gmail.com; 5 Department of Surgery, College of Medicine, Han Yang University, Seoul 04763, Korea; ojkwon@hanyang.ac.kr; 6 Department of Surgery, Korea University Anam Hospital, Seoul 02841, Korea; cwjung@korea.ac.kr; 7 Department of Internal Medicine, Yonsei University College of Medicine, Seoul 03722, Korea; jcyjs@yuhs.ac; 8 Department of Surgery, Yonsei University College of Medicine, Seoul 03722, Korea; ysms91@yuhs.ac; 9 Department of Ophthalmology, CHA Bundang Medical Center, CHA University, Seongnam 13496, Korea

**Keywords:** kidney transplantation, machine learning, risk factors, graft survival

## Abstract

We developed a machine-learning-based model that could predict a decrease in one-year graft function after kidney transplantation, and investigated the risk factors of the decreased function. A total of 4317 cases were included from the Korean Organ Transplant Registry (2014–2019). An XGBoost model was trained to predict the recipient’s one-year estimated glomerular filtration rate (eGFR) below 45 mL/min/1.73 m^2^ using 112 pre- and peri-transplantation variables. The network of model factors was drawn using inter-factor partial correlations and the statistical significance of each factor. The model with seven features achieved an area under the curve of 0.82, sensitivity of 0.73, and specificity of 0.79. The model prediction was associated with five-year graft and rejection-free survival. Post-transplantation hospitalization >25 days and eGFR ≥ 88.0 were the prominent risk and preventive factors, respectively. Donor age and post-transplantation eGFR < 59.8 were connected to multiple risk factors on the network. Therefore, careful donor–recipient matching in older donors, and avoiding pre-transplantation risk factors, would reduce the risk of graft dysfunction. The model might improve long-term graft outcomes by supporting early detection of graft dysfunction, and proactive risk factor control.

## 1. Introduction

Kidney transplantation is the treatment of choice for patients with end-stage kidney disease, and offers improved survival compared with dialysis [1]. Although short-term renal allograft survival has increased substantially over the past three decades, long-term graft survival has remained unsatisfactory [2,3,4]. Therefore, identifying patients at risk of graft loss, and timely intervention, are necessary to prevent late graft failure, and improve long-term graft function [5].

Various factors are associated with late allograft failures, and the serum creatinine level at one year after transplantation can predict long-term renal allograft survival [6,7,8]. Therefore, measuring serum creatinine and estimated glomerular filtration rate (eGFR) is recommended to detect renal allograft dysfunction, according to the KDIGO (Kidney Disease: Improving Global Outcomes) guideline [8]. eGFR is a simple, inexpensive, and universally available method for the assessment of graft function, and can be calculated based on age, sex, and serum creatinine [9].

Notably, prospective observational studies have reported that one-year eGFR is the best predictor of long-term graft function after kidney transplantation [10,11]. Clinical factors associated with eGFR < 65 mL/min/1.73 m^2^ at one-year post-transplantation included an advanced age of the donor and recipient, acute rejection within one year, delayed graft function, deceased donor, and the number of human leukocyte antigen (HLA) mismatches [10]. The risk of late renal allograft failure increased gradually with a lower one-year eGFR, particularly in recipients exhibiting a one-year eGFR of less than 45 mL/min/1.73 m^2^ [11]. Therefore, prediction of the one-year post-transplant eGFR decrease may help to improve long-term graft function and survival.

Machine learning has been used to predict acute rejection, delayed graft function, graft survival, and chronic allograft nephropathy in kidney transplantation, and has advantages over conventional statistics in terms of better performance and the identification of complex associations among predictive factors [12]. A study attempted to predict one-year eGFR in 707 recipients who underwent deceased donor kidney transplantation [13]. The best model for predicting eGFR < 45 mL/min/1.73 m^2^ was a Gaussian support vector machine with recursive feature elimination using five features (donor age, donor death code, recipient weight, recipient height, and recipient sex) from 56 input variables. However, the model performance was unsatisfactory (area under the curve (AUC) = 0.72) [13].

We designed this study to upgrade the model predicting one-year eGFR decline by incorporating up-to-date techniques, such as the extreme gradient boosting (XGBoost) machine learning algorithm [14], automated genetic programming (tree-based pipeline optimization tool, TPOT) for XGBoost hyperparameter tuning [15], and the Boruta algorithm for feature selection [16]. XGBoost enables high-performance boosting decision tree models, and dominates classification problems on structured datasets [17]. Additionally, a factor network was constructed using model-chosen factors, and risk-control targets were explored.

## 2. Materials and Methods

### 2.1. Development and External Validation Data

**Figure 1:**
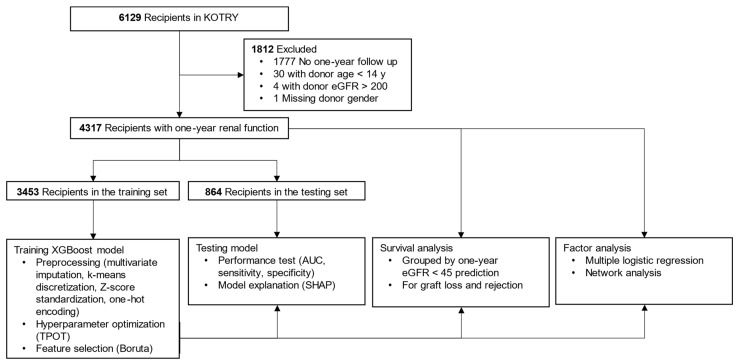
Flow diagram showing data processing and study methods. Abbreviations: KOTRY, Korean Organ Transplant Registry; eGFR, estimated glomerular filtration rate (mL/min/1.73 m^2^); XGBoost, extreme gradient boosting; TPOT, tree-based pipeline optimization tool; AUC, area under the curve; SHAP, shapley additive explanation.

The Korean Organ Transplant Registry (KOTRY) is a prospective multicenter transplantation registry, which includes more than 900 clinical variables [18]. Data of 6129 recipients who underwent kidney transplantation from April 2014 to December 2019 were collected (Figure 1). After excluding 1812 cases with missing data or outliers (donor age under 14 years, donor eGFR over 200 mL/min/1.73 m^2^), a total of 4317 recipients were finally enrolled in this study. All available variables were selected from the recipient and donor in the pre- and peri-transplantation period before postoperative hospital discharge, and donor–recipient relationship variables and immunologic variables (ABO incompatibility, number of HLA mismatches) were extracted (Table 1). We calculated eGFR by the CKD-EPI (Chronic Kidney Disease Epidemiology Collaboration) formula, whereas the Schwartz formula was applied to donors under 19 years old (Supplementary Materials). The model outcome was set as eGFR under 45 mL/min/1.73 m^2^ at one year after kidney transplantation, and the development data were split 8:2 between the training and testing sets, with equal proportions of the model outcome.

Additionally, data of recipients who underwent kidney transplantation between 2010 and 2014, but were not included in the KOTRY cohort, were collected from the Bundang CHA Medical Center for external validation. The study was reviewed and approved by the Institutional Review Board of each transplantation center. The study was conducted following the Declaration of Helsinki, and adhered to the Declaration of Istanbul.

### 2.2. Data Preprocessing: Imputation, Discretization, One-Hot Encoding, and Standardization

Univariate and multivariate imputations were employed for missing categorical and continuous values, respectively. When the skewness or outliers needed to be addressed, the continuous variable was discretized into three categories, low, mid (reference), and high, using discretized k-means clustering (Table 1) [19]. Categorical variables with three or more unique values were one-hot encoded while dropping the reference category, and continuous variables were standardized using z-scores.

### 2.3. Training and Testing of the XGBoost Model

GPU-accelerated TPOT was used to tune nine hyperparameters of XGBoost (Supplementary Materials). Then, a subset of relevant features was identified using the Boruta-SHAP algorithm, and used to train a sparse model (Supplementary Materials). The performance of the sparse model was tested using metrics (AUC, sensitivity, specificity), and explained using Shapley additive explanation (SHAP) values in the test set [20]. A threshold was the Youden index for the training set, which was applied in testing and validation.

### 2.4. Survival Analysis of the Model Prediction

Time-to-event analyses on death-censored graft loss and biopsy-proven rejection were performed according to the model prediction for the right-censored development data (Supplementary Materials). The total numbers of cases were 4305 and 4317 for graft and rejection-free survival, respectively. Twelve patients who experienced graft loss within one year were excluded from the graft survival analysis.

### 2.5. Statistical and Network Analyses of Model Factors

The statistical significance of the model or known factors was calculated using multiple logistic regression for the development data. In addition to model factors, the age and sex of the donor and recipient were included as covariates.

A partial correlation matrix of factors was then generated, and a thresholded extended Bayesian information criterium (EBIC) graphical lasso network was plotted (Supplementary Materials). The network was simplified by excluding spurious connections using graphical lasso regularization with EBIC model selection [21]. The node size of the factor was calculated using the effective size of the odds ratio, and each edge weight reflected the correlation strength between two nodes after controlling all other network correlations.

### 2.6. Statistics and Software

Two programming languages were used: Python (version 3.7.10, Python Software Foundation, Wilmington, DE, USA) for preprocessing, machine learning, and survival analysis; and R (3.6.3, R Foundation for Statistical Computing, Vienna, Austria) for multiple logistic regression and network analysis. DeLong’s confidence intervals of AUC were calculated using the pROC package of R [22]. Detailed software usage is described in Supplementary Materials.

## 3. Results

### 3.1. Selected Model Features

The mean one-year eGFR was 70 (SD 20) (95% CI, 69–70) mL/min/1.73 m^2^, and the proportion of recipients exhibiting one-year eGFR < 45 mL/min/1.73 m^2^ was 11% (378/3453) in the training set. Hyperparameter optimization of XGBoost was attempted with 112 variables, and none of the variables were dropped by the regularization of XGBoost. However, a sparse model with seven variables (6% of total variables), which included donor and recipient age, low eGFR at discharge, high eGFR at discharge, serum creatinine at discharge, post-transplantation stay, and height difference between donors and recipients, was built by feature selection using the Boruta algorithm.

### 3.2. Model Performance and Explanation

**Figure 2:**
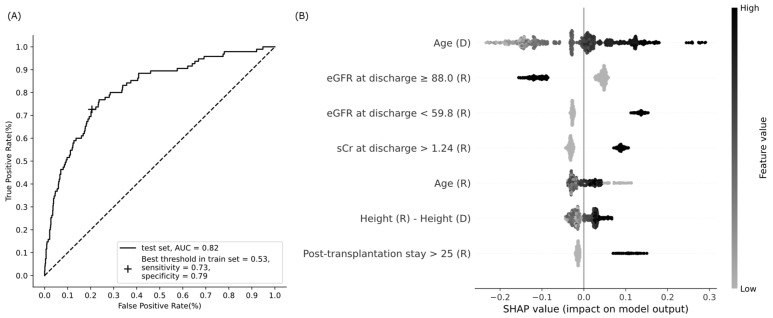
Performance of the XGBoost model to predict a decline in eGFR at one year after kidney transplantation. (**A**) Receiver operating characteristic curve; (**B**) summary plot of Shapley additive explanations (SHAP). Model features are sorted along the y axis of the summary plot by the sum of SHAP value (probability) magnitudes over all cases in the test data, and the distribution of each feature’s impacts is plotted using SHAP values of individual cases. Binary features have 1 if present, or 0 if absent. Abbreviations: AUC, area under the curve; eGFR, estimated glomerular filtration rate (mL/min/1.73 m^2^); sCr, serum creatinine (mg/dL); D, donor; R, recipient.

The prediction model with seven variables showed a moderate performance, with an AUC of 0.82 (95% CI, 0.77–0.86, DeLong), sensitivity of 0.73, and specificity of 0.79 on the test data (Figure 2). External validation with 51 recipients showed similar performance, with an AUC of 0.83, sensitivity of 0.76 (13/17), and specificity of 0.71 (24/34). According to the SHAP values, donor age had the most significant impact, and eGFR at discharge ranked second (Figure 2). Several factors had elongated tails in the SHAP summary plot, specifically, an elderly donor, eGFR at discharge <59.8 mL/min/1.73 m^2^, and a post-transplantation hospitalization longer than 25 days increased the probability of a decline in one-year eGFR up to 0.29, 0.15, and 0.15, respectively (Figure 2). A group of high SHAP probabilities for donor age was found on the plot with a median age of 72 (range, 70–81) years and their median SHAP value of 0.27 (range, 0.24–0.29) (Figure 2). By contrast, a young donor and eGFR at discharge ≥88 mL/min/1.73 m^2^ reduced the probability up to 0.23 and 0.16, respectively (Figure 2). Notably, the SHAP value distribution of the recipient age was non-linear; the probability increased in both high (over 57 years) and low (less than 29 years) recipient ages (Figure 2).

### 3.3. Graft and Rejection-Free Survival for Model Prediction

**Figure 3:**
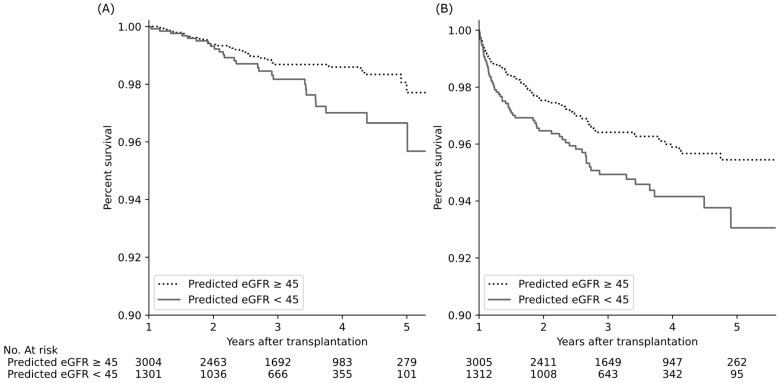
Kidney transplant outcomes according to predicted one-year eGFR levels of the recipient. Predicted eGFR decline was associated with a significant decrease in graft survival (**A**) and increase in cumulative incidence of biopsy-proven rejection (**B**) (adjusted hazard ratios of 1.9 and 1.6, *p* = 0.02 and *p* = 0.007, respectively, Cox proportional hazard regression with age and sex). Abbreviations: eGFR, estimated glomerular filtration rate (mL/min/1.73 m^2^).

The graft survival rate was significantly worse, and the cumulative incidence of rejection was significantly higher in recipients with predicted eGFR < 45 mL/min/1.73 m^2^ than in those with predicted eGFR > 45 mL/min/1.73 m^2^ (adjusted hazard ratios of 1.9 and 1.6, *p* = 0.02 and 0.007, respectively; Figure 3).

### 3.4. Statistical Significance of Model Features

All seven model factors had a statistically significant association with a decline in one-year eGFR (Table 2). Both low and high recipient ages were significantly associated with reduced one-year eGFR when the recipient age was categorized into low (19–28 years), medium (29–57 years, reference), and high (58–76 years) groups according to SHAP value distribution (Figure 2 and Table 2).

Additionally, being a female recipient was a risk factor when the recipient’s sex was added as a covariate (Table 2). Among the reported non-model factors, that is, deceased donor, delayed graft function, and the number of HLA mismatches, the number of HLA mismatches remained a significant factor (Table 2) [10].

### 3.5. Network Analysis of Model Predictors

**Figure 4:**
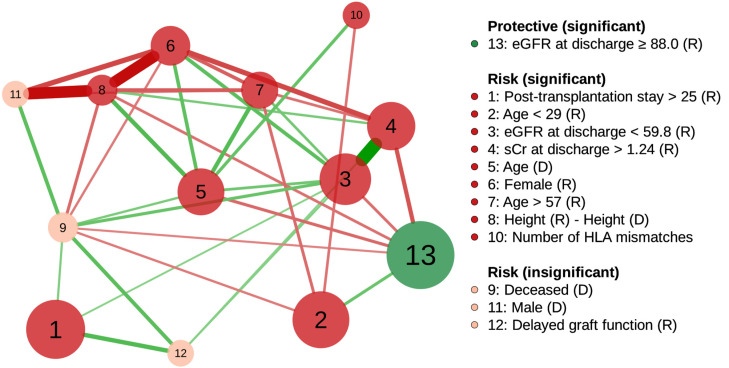
Partial correlation network of factors associated with one-year renal allograft dysfunction using graphical lasso regularization. Node size is proportional to the effective size of the odds ratio. The nodes are positively (risk) or negatively (protective) related to graft function decline. Green and red edges represent positive and negative correlations between the nodes, respectively. The edge with the highest absolute weight has full-color saturation and the widest width. Abbreviations: R, recipient; D, donor; eGFR, estimated glomerular filtration rate (mL/min/1.73 m^2^); sCr, serum creatinine (mg/dL); HLA, human leukocyte antigen.

The largest node was “eGFR at discharge ≥88.0”, and the most prominent risk node was “Post-transplantation stay >25” (Figure 4). In addition, two risk nodes could build up the risk effect together with other factors. The node with the highest potential was “eGFR at discharge <59.8”, which was positively connected to five risk nodes: “Post-transplantation stay >25”, “Serum creatinine at discharge >1.24”, “Donor age”, “Female recipient”, and “Recipient age > 57”, and negatively connected to a preventive factor, “eGFR at discharge ≥88.0”. Second, “Donor age” was positively associated with four risk nodes: “eGFR at discharge <59.8”, “Female recipient”, “Recipient age > 57”, and “Donor-recipient height difference”, and negatively associated with a preventive node, “eGFR at discharge ≥88.0”. Consequently, adjusted odds ratios of “eGFR at discharge <59.8” and “Donor age” were 2.1 and 1.9, respectively (multiple logistic regression, Table 2), but the crude odds ratios increased to 4.7 (95% CI, 3.8–5.8, 59.8–88.0 mL/min/1.73 m^2^ as the reference) and 2.6 (95% CI 2.3–2.9), respectively, by the effect of associated risk factors (univariate logistic regression).

Two insignificant risk nodes, “Deceased donor” and “Delayed graft function”, were simultaneously connected to two significant risk nodes: “Post-transplantation stay >25” and “eGFR at discharge <59.8” (Figure 4). “Deceased donor” without “eGFR at discharge <59.8”, and “Delayed graft function” without “eGFR at discharge <59.8” and “Post-transplantation stay >25” were statistically significant (*p* = 0.03 and *p* = 0.049, respectively; multiple logistic regression).

## 4. Discussion

### 4.1. Principal Findings

One-year eGFR < 45 mL/min/1.73 m^2^ was successfully predicted using seven factors (donor and recipient ages, low and high eGFR levels after transplantation, high serum creatinine after transplantation, height difference between the donor and recipient, and post-transplantation stay), and the predicted decline in one-year eGFR was related to long-term allograft outcomes. Two risk factors, donor age and low eGFR after transplantation, were noticeable on the network because of their multiple positive connections to other risk factors.

### 4.2. Improving the Performance of the XGBoost Model

A higher performance (AUC = 0.8) of the model was obtained when predicting the one-year eGFR < 45 mL/min/1.73 m^2^ than in the previous report (AUC = 0.7) [13]; however, the current and previous studies could not be directly compared because of the difference in development data size (4317 vs. 707), transplantation period (2014–2019 vs. 1998–2008), ethnicity (Asian vs. European), donor type (living and deceased vs. deceased only), and machine learning type (classifier vs. regressor). However, the following techniques were used to improve the model performance.

#### 4.2.1. Automated Machine Learning

A developer configures the XGBoost hyperparameters to obtain the best performance, and the results can vary according to the developer’s experience [17]. This study used the GPU-accelerated TPOT, one of the most effective automated machine learning tools. TPOT uses genetic algorithms to find the best combination of hyperparameters, and GPUs increase the chance that TPOT finds more advanced combinations by accelerating its computation time [23].

#### 4.2.2. Addressing the Imbalanced Classification Problem

The minority class is more difficult to predict because a model may focus on learning the characteristics of the abundant cases from the majority class [24]. This study had a moderate imbalance problem of 11% of the one-year eGFR < 45 mL/min/1.73 m^2^ (positive class), which was addressed using class-weighted XGBoost [24]. In detail, the model was eight times more likely to correct errors in the positive class, and successfully achieved balanced sensitivity and specificity.

### 4.3. Clinical Relevance of Discretized Factors

Some numerical variables were discretized to categorical variables, and the data-driven discretization criteria had clinical relevance. For example, eGFR < 59.8 and ≥ 88.0 mL/min/1.73 m^2^ correspond to GFR categories in chronic kidney disease, that is, “mildly to moderately decreased” (GFR 45–59, G3 or higher category) and “normal or high” (GFR ≥ 90, G1 category), respectively [25]. For post-transplantation stay >25 days, longer than 14 days is a risk factor of five-year mortality, and longer than 30 days was reported in 1% of recipients [26].

### 4.4. Factors Associated with a Decline in One-Year Renal Allograft Function

#### 4.4.1. Donor Age, the Most Influential Factor in the Model Prediction

Donor age has been reported as the most critical factor governing graft survival and patient mortality after kidney transplantation [1]. “Donor age” had long tails in low and high SHAP values on the summary plot, which meant that the probability varied considerably with age (Figure 2). Age > 70 years required special attention because of a dramatic increase in the probability of one-year eGFR decrease in this group (Figure 2), and this finding has been consistently demonstrated [27,28,29].

#### 4.4.2. eGFR at Discharge, the Most Statistically Significant Factor

The factor eGFR at discharge ≥88.0 mL/min/1.73 m^2^ showed the strongest association with a one-year eGFR decrease, and eGFR at discharge <59.8 mL/min/1.73 m^2^ was a potent risk factor (Table 2 and Figure 2). The KDIGO guideline recommends daily measurement of serum creatinine, and calculation of eGFR until hospital discharge after kidney transplantation to detect acute changes in renal allograft function, such as acute rejection, obstruction, urine leak, vascular compromise, and recurrent infection diseases, which are more common early after transplantation [30]. 

#### 4.4.3. Other Significant Model Factors

“Post-transplantation stay >25” was a significant risk node, and positively connected to “eGFR at discharge <59.8” on the factor network, which means that hospitalization length might be associated with early renal allograft function (Figure 2 and Figure 4). Prolonged hospitalization can be caused by early renal allograft dysfunction, and patients with poor fitness or health for transplantation tend to have an extended length of stay during transplant admission [1,30].

A tall recipient for a short donor was another significant risk factor (Table 2 and Figure 2). Since body height is correlated with kidney length, the results imply that a relatively small donor kidney may not be sufficient for a tall recipient [31,32]. An explanation for the kidney size mismatch problem is nephron dosing, suggesting that the graft size should match the recipient’s physiologic demand to prevent renal damage by glomerular hyperfiltration.

A recipient age under 29 years or over 57 years was significantly related to a decline in one-year eGFR (Table 2). A recipient age ≥ 60 years is a risk factor for one-year eGFR decline, and affects the five-year eGFR by lowering one-year eGFR [10]. In addition, previous studies have demonstrated that renal allograft survival is the worst in young recipients between 10 and 20 years of age [33,34]. It should also be noted that the proposed network showed that the recipient age < 29 years was associated with high eGFR at discharge, whereas the recipient age > 57 years was related to low eGFR at discharge (Figure 4). Accordingly, low and high recipient ages would have different reasons for a one-year eGFR drop. The increased risk of graft failure in young adults is partly explained by non-adherence to immunosuppressive medications, and vulnerability to graft rejection [35,36].

#### 4.4.4. Significant Non-Model Factors

Two significant non-model factors, the female recipient and the number of HLA mismatches, were included as a covariate and known factor, respectively. Female recipients show higher graft failure risks than male recipients, especially when their donors are male, presumably because of sex-determined minor histocompatibility antigens, or the influence of sex hormones on immune activation [37,38]. HLA mismatch is a known risk factor for low one-year eGFR [10] and graft loss one-year post-transplantation [39].

### 4.5. Application of Factor Network for Finding Control Targets and Confounders

Two significant risk nodes, “eGFR at discharge <59.8” and “Donor age”, had multiple positive connections to other risk factors in the network (Figure 4). These connections explained the increase in the factors’ unadjusted odds ratios to 2.2 and 1.4 times their adjusted odds ratios, respectively. Therefore, controlling these two risk factors might considerably reduce the risk of declining one-year eGFR.

For “eGFR at discharge <59.8”, there might be a causal relationship with pre-transplantation risk factors, such as “Donor age”, “Female recipient”, and “Recipient age > 57”, which occurred temporally before “eGFR at discharge <59.8” (Figure 4). For example, a graft from an older donor reduced the number of viable nephrons, and led to early graft dysfunction [40].

“Donor age” was positively connected to “Female recipient”, “Recipient age > 57”, “Height(R)−Height(D)”, and “Number of HLA mismatches”, which means that care should be taken when matching a recipient with an older donor (Figure 4). For example, better HLA matching offsets the disadvantages of the older donor in deceased donor kidney transplantation [41]. Lastly, the positive connection between “Donor age” and “eGFR at discharge <59.8” should be emphasized because donor age could affect the one-year eGFR and long-term outcome through low eGFR at discharge (Figure 3 and Figure 4).

The factor network revealed that two known risk nodes, “Deceased donor” and “Delayed graft function”, could be confounded by “eGFR at discharge <59.8” and “Post-transplantation stay >25” through their positive connections (Figure 4). For a deceased donor, a prolonged ischemia–reperfusion injury is usually an inevitable event affecting early kidney allograft function [42]. The injury also causes delayed graft function (see the positive connection between nodes 9 and 12 in Figure 4), which is reported to be associated with a decreased eGFR at discharge, and increased length of stay after transplantation [26,43].

### 4.6. Limitations

First, the relationships among the factors on the network should be considered associative, not causal. However, causal relationships between factors can be suggested if they had temporality and plausibility [44]. Second, the results may change because of the different ethnicities of the recipients and donors, and the discharge policies after transplantation. Third, there might be unfound pre-, peri-, and post-transplantation factors, such as medication adherence, that would significantly alter the one-year eGFR [13].

## 5. Conclusions

An XGBoost-based model successfully predicted the one-year eGFR reduction in kidney transplant recipients with good performance. In addition to the reported factors, novel pre- and peri-transplantation factors were discovered by selecting all relevant features from nationwide cohort data. Donor age and eGFR at discharge had the highest impact on model prediction, and could be primary risk factor control targets. Therefore, this study could help control one-year eGFR-associated risk factors before and after kidney transplantation, and allow physicians to plan preemptive measures when the graft function would be compromised. A practice using the proposed model and network could provide clinical benefits, as model-predicted graft dysfunction is related to long-term graft survival and rejection.

## Figures and Tables

**Table 1:** Study variables.

Recipient Variables
**General characteristics** age, sex, BMI, smoking, primary renal disease, dialysis vintage, dialysis type, transplantation history, medication ^a^
**Comorbidities** diabetes, hypertension, cardiovascular disease, tumor
**Laboratory findings** WBC count, neutrophil count (%), hemoglobin, hematocrit, platelet, BUN, creatinine, eGFR, uric acid, albumin, calcium ^b^, phosphorus, fasting serum glucose, hsCRP ^c^, PTH level ^c^, total cholesterol, LDL, HDL, triglyceride
**Immunology and immunosuppression** ABO incompatibility; T and B cell crossmatch; HLA A, B, DR mismatch numbers; donor-specific antibody; desensitization; induction immunosuppression ^d^; maintenance immunosuppression ^e^
**Perioperative findings** delayed graft function, creatinine at discharge ^c^, eGFR at discharge ^c^, post-transplantation stay ^c^
**Donor Variables**
**General characteristics** age, sex, BMI, smoking, donor type ^f^, cold ischemic time ^c^, donation after cardiac death
**Comorbidities** diabetes, hypertension, cardiovascular disease, tumor
**Laboratory findings** WBC count ^c^, neutrophil count (%) ^c^, hemoglobin ^c^, hematocrit ^c^, platelet ^c^, BUN ^c^, creatinine ^c^, eGFR ^c^, uric acid ^c^, albumin ^c^, calcium ^b,c^, phosphorus ^c^, fasting serum glucose ^c^, hsCRP ^c^, total cholesterol ^c^, proteinuria
**Donor–recipient Relationship Variables**
age difference, sex match, height difference, weight difference, BSA ratio, viral serostatus ^g^

^a^ Aspirin, statin, or vitamin D analog. ^b^ Corrected calcium = total calcium + 0.8 × (4 − albumin), where calcium in mg/dL, and albumin in g/dL. ^c^ Discretized to low, mid, high categories. ^d^ Basiliximab, anti-thymocyte globulin, or both. ^e^ Steroid, tacrolimus, cyclosporine, mycophenolate mofetil. ^f^ Deceased or living. ^g^ For hepatitis B virus, positive if donor hepatitis B surface antigen (HBsAg) is positive and recipient hepatitis B surface antibody (HbsAb) is negative. For hepatitis C virus, Epstein–Barr virus, and cytomegalovirus, positive if donor IgG serostatus is positive and recipient IgG serostatus is negative. Abbreviations: BMI, body mass index; WBC, white blood cell; HLA, human leukocyte antigen; BUN, blood urea nitrogen; eGFR, estimated glomerular filtration rate; hsCRP, high-sensitivity c-reactive protein; PTH, parathyroid hormone; LDL, low-density lipoprotein; HDL, high-density lipoprotein; BSA, body surface area.

**Table 2:** Factor analysis for one-year renal allograft dysfunction (multiple logistic regression).

	eGFR ≥ 45 (N = 3453)	eGFR < 45 (N = 864)	OR (95% CI)	*p* Value
**Categorical Factors**	n (%)	n (%)		
eGFR (mL/min/1.73 m^2^) ^a^				
<59.8	871 (23%)	315 (67%)	2.1 (1.5–2.9)	<0.001
59.8–88.0	1725 (45%)	133 (28%)	NA	NA
≥88.0	1248 (32%)	25 (5.3%)	0.4 (0.2–0.6)	<0.001
Recipient age (year) ^b^				
<29	214 (5.6%)	29 (6.1%)	2.3 (1.5–3.7)	<0.001
29–57	2755 (72%)	263 (56%)	NA	NA
>57	875 (23%)	181 (38%)	1.5 (1.2–1.9)	<0.001
Post-transplantation stay (day) ^a^				
≤25	3501 (91%)	354 (75%)	NA	NA
>25	343 (8.9%)	119 (25%)	2.4 (1.8–3.2)	<0.001
Serum creatinine (mg/dL) ^a^				
≤1.24	2787 (73%)	151 (32%)	NA	NA
>1.24	1057 (27%)	322 (68%)	2.0 (1.4–2.8)	<0.001
Female recipient ^c^	1602 (42%)	162 (34%)	1.6 (1.2–2.2)	0.002
Deceased donor ^d^	1331 (35%)	253 (53%)	1.2 (1.0–1.6)	0.08
Male donor ^c^	2064 (54%)	238 (50%)	1.1 (0.8–1.5)	0.49
Delayed graft function ^d^	116 (3.0%)	43 (9.1%)	1.1 (0.7–1.7)	0.68
**Continuous Factors**	mean (SE)	mean (SE)		
Donor Age (year)	46 (0.2)	56 (0.5)	1.9 (1.7–2.2) ^e^	<0.001
Height (R)−Height (D) (cm)	−1 (0.2)	3 (0.6)	1.3 (1.1–1.5) ^e^	0.007
HLA mismatch numbers ^d^ (range 0–6)	3.2 (0.03)	3.6 (0.07)	1.1 (1.0–1.3) ^e^	0.04

^a^ Measured at discharge in recipients. ^b^ Not significant (*p* = 0.39) when a continuous factor. ^c^ Not model factors, but included to control sex effect. ^d^ Not model factors, but reported risk factors [10]. ^e^ Standardized odds ratio. Abbreviations: eGFR, estimated glomerular filtration rate; OR, odds ratio; CI, confidence intervals; SE, standard error; R, recipient; D, donor; HLA, human leukocyte antigen; NA, not available for a reference category.

## Data Availability

Data are available upon reasonable research proposal to the Korean Organ Transplantation Registry (https://www.kotry.org/eng/, (accessed on 24 February 2022)).

## Supplementary Materials

The following supporting information can be downloaded at: https://www.mdpi.com/article/10.3390/jcm11051259/s1; eGFR calculation, TPOT, XGBoos, feature selection using the Boruta algorithm, survival analysis, multiple logistic regression, network graph, and Scikit-learn tools [14,15,16,17,45,46,47,48,49,50].
