# Supplementary material for: Personalized Prediction of Kidney Function Decline and Network Analysis of the Risk Factors after Kidney Transplantation Using Nationwide Cohort Data
Source: J Clin Med. 2022 Feb 25;11(5):1259. doi: 10.3390/jcm11051259 (PMC8911006; doi:10.3390/jcm11051259)
Supplement: Supplementary file 1 [file jcm-11-01259-s001.zip › jcm-1602664-supplementary.pdf]

## Supplementary Materials

### 1. eGFR Calculation

The CKD-EPI (Chronic Kidney Disease Epidemiology Collaboration) formula was used to calculate eGFR except for donors under 19 years old [1,2]. CKD-EPI eGFR = 141 × minimum(sCr/κ, 1)$^{\alpha}$× maximum(sCr/κ, 1)$^{-1.209}$× 0.993$^{age}$× 1.018 (if female), where κ is 0.7 for females and 0.9 for males, $\alpha$ is −0.329 for females and −0.411 for males, and sCr is serum creatinine (mg/dL). The Schwartz formula was applied to donors under 19 years old, in which eGFR = 0.413 × (Height/sCr), where height is in centimeters, and sCr is serum creatinine (mg/dL).

### 2. TPOT

TPOT builds 100 random models, and chooses the best model using cross-validation. Then, genetic programming, for example, offspring crossover or random mutation, is used to alter the chosen model's hyperparameters. This evaluate–select–crossover–mutate process was repeated through 100 generations, and the model with the best set of hyperparameters was selected [3]. The following hyperparameters of XGBClassifier were tuned by TPOT: the number of boosting rounds ("n_estimators"), boosting learning rate ("learning_rate"), maximum tree depth for base learners ("max_depth"), minimum sum of instance weight needed in child ("min_child_weight"), minimum loss reduction required to make new branching from a node ("gamma"), subsample ratio of the training instance ("subsample"), subsample ratio of columns ("colsample_bytree"), L1 regularization term ("reg_alpha"), and L2 regularization term ("reg_lambda"). The hyperparameters can be classified into four subgroups: tree constraints (tree numbers, tree depth, minimum leaf weights, minimum improvement to loss), shrinkage (learning rate), random sampling (subsampling rows and columns), and penalized learning (L1 and L2 regularization) [4].

To control the imbalance between positive and negative classes for the model outcome, "scale_pos_weight" was set as the ratio of the number of negative cases to the number of positive cases. The parameter "objective" was set to "binary:logistic," logistic regression for binary classification. Then, TPOTClassifier evaluated models using AUC ("roc_auc"). The number of iterations to run the classifier optimization process ("generations"), and the number of models in each iteration ("population_size") of the TPOTClassifier, were set to 100.

### 3. Extreme Gradient Boosting (XGBoost) and Feature Selection Using the Boruta Algorithm

Gradient boosting algorithms build multiple decision trees to exploit data, and XGBoost is one implementation [5]. With the best hyperparameter set for the XGBoost classifier found by TPOT, the Boruta-SHAP library for Python was applied to feature selection using the following process [6,7]: (1) Create and append randomly permutated features, that is, shadow features, to original data. (2) Calculate the feature importance in the SHAP value of original and shadow features. (3) Repeat (1) and (2) for 100 iterations. From multiple measurements, distributions of SHAP for each feature are estimated. (4) Compare SHAP values of an original feature and its shadow feature. The original feature is selected if its mean SHAP value is statistically greater than the mean value of the shadow feature (t-test). Then, the final model is trained with selected features, and the model AUC is compared with the AUC of the model before feature selection.

### 4. Survival Analysis

We predicted eGFR < 45 mL/min/1.73 m$^2$ of 1-year renal allograft for each case of the development data, and performed leave-one-out cross-validation using the "LeaveOneOut" class in scikit-learn tools [8].

During the leave-one-out cross-validation, each sample was used once as a test case, whereas the remaining samples formed the training set [9]. We used "KaplanMeierFitter" and "CoxPHFitter" of lifelines packages for Python to fit the Kaplan–Meier estimate for the survival function and Cox's proportional hazard model, respectively.

## 5. Multiple Logistic Regression and Network Graph

We performed multiple logistic regression using the "glm" function of R with "binomial" for the "family" argument. For network analysis, we calculated a correlation matrix using the "cor" function of the R stats package with "pearson" for the method argument, and drew a network graph using the "qgraph" function on the qgraph package of R with "glasso" and "spring" for the graph and layout arguments, respectively [10]. The node size was proportional to the odds ratio if the node was a risk factor, and 1/odds ratio if it was a protective factor.

## 6. Scikit-learn Tools

We used the following scikit-learn tools: "IterativeImputer" class with the "ExtraTreesRegressor" estimator for multivariate imputation; "KBinsDiscretizer" class to discretize a variable into three bins where values have the same nearest center of a 1D k-means cluster in each bin; "StandardScaler" for standardization; "roc_curve" and "roc_auc_score" for sensitivity, specificity, threshold, and AUC calculations [8].

## Supplementary References


1. Levey, A.S.; Stevens, L.A.; Schmid, C.H.; Zhang, Y.; Castro III, A.F.; Feldman, H.I.; Kusek, J.W.; Eggers, P.; Van Lente, F.; Greene, T. A new equation to estimate glomerular filtration rate. *Annals of internal medicine* **2009**, *150*, 604-612.
2. Schwartz, G.J.; Munoz, A.; Schneider, M.F.; Mak, R.H.; Kaskel, F.; Warady, B.A.; Furth, S.L. New equations to estimate GFR in children with CKD. *Journal of the American Society of Nephrology* **2009**, *20*, 629-637.
3. Olson, R.S.; Moore, J.H. TPOT: A Tree-Based Pipeline Optimization Tool for Automating Machine Learning. In *Automated Machine Learning*, Hutter, F., Kotthoff, L., Vanschoren, J., Eds.; The Springer Series on Challenges in Machine Learning; Springer: Cham, 2019; Volume 17, pp. 151–160.
4. Brownlee, J. *XGBoost With Python: Gradient Boosted Trees with XGBoost and scikit-learn*; Machine Learning Mastery: 2018.
5. Chen, T.; Guestrin, C. XGBoost: A Scalable Tree Boosting System. *In: KDD '16: Proceedings of the 22nd ACM SIGKDD International Conference on Knowledge Discovery and Data Mining. New York, NY: Association for Computing Machinery* **2016**, 785–794, doi:10.1145/2939672.2939785.
6. Kursa, M.B.; Rudnicki, W.R. Feature Selection with theBorutaPackage. *Journal of Statistical Software* **2010**, *36*, doi:10.18637/jss.v036.i11.
7. Keany, E. BorutaShap 1.0.15. Available online: https://pypi.org/project/BorutaShap (accessed on 1 February 2022).
8. Pedregosa, F.; Varoquaux, G.; Gramfort, A.; Michel, V.; Thirion, B.; Grisel, O.; Blondel, M.; Prettenhofer, P.; Weiss, R.; Dubourg, V. Scikit-learn: Machine learning in Python. *the Journal of machine Learning research* **2011**, *12*, 2825-2830.
9. Lachenbruch, P.A.; Mickey, M.R. Estimation of error rates in discriminant analysis. *Technometrics* **1968**, *10*, 1-11.
10. Epskamp, S.; Cramer, A.O.; Waldorp, L.J.; Schmittmann, V.D.; Borsboom, D. qgraph: Network visualizations of relationships in psychometric data. *Journal of statistical software* **2012**, *48*, 1-18.
